# IL-28 Supplants Requirement for T_reg_ Cells in Protein σ1-Mediated Protection against Murine Experimental Autoimmune Encephalomyelitis (EAE)

**DOI:** 10.1371/journal.pone.0008720

**Published:** 2010-01-14

**Authors:** Agnieszka Rynda, Massimo Maddaloni, Javier Ochoa-Repáraz, Gayle Callis, David W. Pascual

**Affiliations:** 1 Veterinary Molecular Biology, Montana State University, Bozeman, Montana, United States of America; 2 Department of Microbiology and Immunology, Dartmouth Medical School, Lebanon, New Hampshire, United States of America; New York University, United States of America

## Abstract

Conventional methods to induce tolerance in humans have met with limited success. Hence, efforts to redirect tolerogen uptake using reovirus adhesin, protein sigma 1 (pσ1), may circumvent these shortcomings based upon the recent finding that when reovirus pσ1 is engineered to deliver chicken ovalbumin (OVA) mucosally, tolerance is obtained, even with a single dose. To test whether single-dose tolerance can be induced to treat EAE, proteolipid protein (PLP_130–151_) was genetically fused to OVA to pσ1 (PLP:OVA-pσ1) and shown to significantly ameliorate EAE, suppressing proinflammatory cytokines by IL-10^+^ forkhead box P3 (FoxP3)^+^ CD25^+^CD4^+^ T_reg_ and IL-4^+^CD25^−^CD4^+^ Th2 cells. IL-10R or IL-4 neutralization reversed protection to EAE conferred by PLP:OVA-pσ1, and adoptive transfer of Ag-specific T_reg_ or Th2 cells restored protection against EAE in recipients. Upon assessment of each relative participant, functional inactivation of CD25 impaired PLP:OVA-pσ1's protective capacity, triggering TGF-β-mediated inflammation; however, concomitant inactivation of TGF-β and CD25 reestablished PLP:OVA-pσ1-mediated protection by IL-28-producing FoxP3^+^CD25^−^CD4^+^ T cells. Thus, pσ1-based therapy can resolve EAE independently of or dependently upon CD25 and assigns IL-28 as an alternative therapy for autoimmunity.

## Introduction

Th17 cells are pivotal for EAE pathogenesis [1], although reversible by regulatory cell intervention, including FoxP3^+^ CD25^+^CD4^+^ T (T_reg_) cells producing IL-10 [2]–[5] and/or TGF-β [3], [6]–[8]. Recently, IL-13-producing T_reg_ cells induced by an oral recombinant *Salmonella* vaccine exhibiting anti-encephalitogenic properties have also been found to treat EAE [8]. Anti-inflammatory Th2 cells, traditionally viewed secondary to T_reg_ cells, can enhance recovery from EAE and lessen EAE when adoptively transferred into diseased animals [7], [8]. Although the Th2-type IL-4 cytokine can trigger T_reg_ cell responses [4], [5], [7], [9], it can compromise T_reg_ cell-mediated suppression of asthma [10], suggesting that Th2 cells and/or their cytokines are important regulators of immunosuppression.

Secreted by dendritic cells (DCs) and macrophages, IL-28B (IFNλ 3) [11]–[13], a newly described member of IFNλ family, is known for its anti-inflammatory activity [14]. Sharing a common signaling pathway with anti-viral type I IFNs [12], IL-28's role in EAE has yet to be evaluated, but it can prime tolerogenic DCs in vitro [12]. When adapted as an adjuvant during DNA vaccination, plasmid-encoded IL-28B reduces T_reg_ cell numbers, but enhances granular CD8^+^ T cells [15]. In this current study, we demonstrate that protection against EAE, mediated by pσ1, is conferred by the expected IL-10-producing T_reg_ cells; however, in the absence of functional T_reg_ cells, protection is mediated by IL-28-producing Th2 cells, demonstrating for the first time that Th2 cells produce IL-28, and endogenous IL-28 can confer protection against EAE.

Acquisition of responsiveness to myelin proteins can develop into the autoimmune disorder, multiple sclerosis (MS) [16]. Current MS therapies fail to restore the unresponsiveness to these self-antigens (Ags). While feeding myelin Ags is effective against EAE [3], [17]–[19], when applied to patients, oral feeding with bovine myelin preparations was deemed unsuccessful [20]. Thus, conventional methods to elicit oral tolerance need to be improved. A number of studies have sought to enhance induction of oral tolerance by adapting liposome delivery [21], including oral adjuvants [22] or coupling to mucosal binding molecules [23], [24]. Although most of these strategies significantly ameliorate EAE, multiple doses are required to sustain tolerance, lessening the potency of such methods.

Past studies suggest that sustainable tolerance requires the presence of Peyer's patches for initial Ag sampling subsequent oral tolerogen ingestion [25]. Such evidence implicates the importance of Ag-sampling microfold (M) cells to facilitate Ag uptake from the lumenal surface. To direct tolerogen uptake, we hypothesized that M cell adhesins could be employed to target mucosal inductive tissues, as readily induced with a single dose of OVA fused to reovirus pσ1, [5], [26]. Mucosal OVA-pσ1 induced Ag-specific IL-10^+^ T_reg_ and IL-4^+^ Th2 cells capable of suppressing immunity to OVA and pσ1, even when co-administered with potent mucosal adjuvants [5], [26].

Noting the potency of pσ1-elicited tolerance, we queried if it could be adapted to treat autoimmunity by genetically fusing two copies of a portion of proteolipid protein (PLP) containing the encephalitogenic sequence (PLP_139–151_) to OVA-pσ1, termed PLP:OVA-pσ1. The described studies showed that PLP_139–151_-induced EAE is ameliorated with a single nasal dose of PLP:OVA-pσ1, stimulating the induction of IL-10-producing T_reg_ cells and IL-4-producing FoxP3^+^ Th2 cells. Notwithstanding that these induced regulatory T cells were entirely protective subsequent their adoptive transfer, and their effects were neutralized by anti-IL-10 receptor (IL-10R) or anti-IL-4 mAb, additional analyses sought to assess alternative regulatory T cell pathways. Functional inactivation of PLP:OVA-pσ1-primed T_reg_ cells rendered mice to an aggressive EAE driven by TGF-β-induced Th17 cells. However, PLP:OVA-pσ1 could re-confer protection against EAE upon CD25 and TGF-β co-neutralization in a reversible, IL-28-dependent fashion. Thus, these results show that pσ1-based therapeutics can stimulate multiple pathways to induce tolerance and, importantly, can be accomplished independently of T_reg_ cells via IL-4^+^ or IL-28^+^ Th2 cells.

## Results

### Nasal PLP:OVA-pσ1 Ameliorates EAE

**Figure 1:**
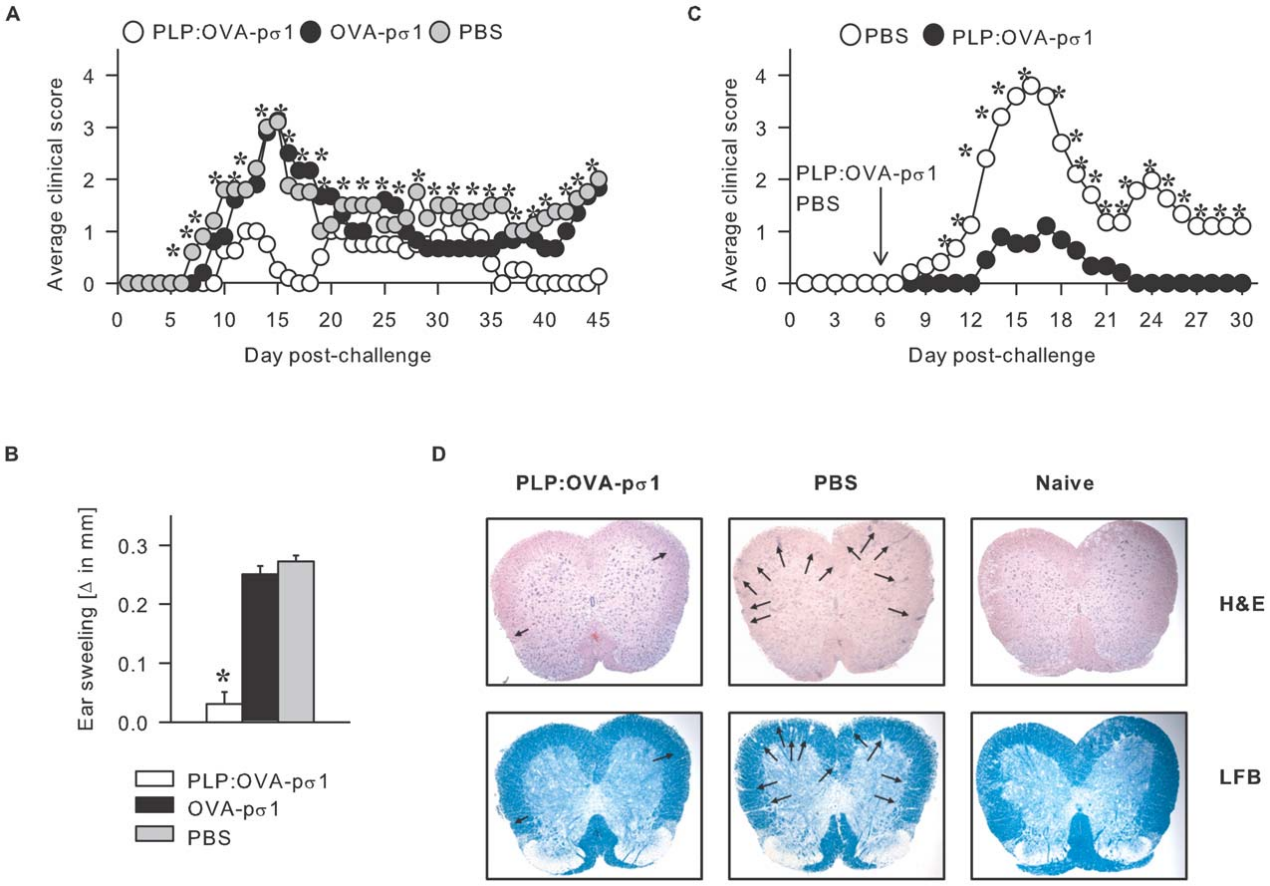
Nasal administration of PLP:OVA-pσ1 protects mice from EAE. **A.** Mice were dosed with 100 µg of PLP:OVA-pσ1, OVA-pσ1, or PBS on days −21, −14, and −7, challenged with PLP_139–151_ peptide on day 0, and monitored daily for the development of clinical disease. PBS and OVA-pσ1-dosed mice developed fully pronounced EAE and never recovered completely. PLP:OVA-pσ1-immunization prior to EAE induction significantly delayed and ameliorated clinical onset of EAE and resulted in a complete recovery of all PLP:OVA-pσ1-dosed mice. Average of 10–15 mice per group is shown. * P<0.05 for PLP:OVA-pσ1 vs. PBS. **B.** Anti-PLP_139–151_ DTH response measured two weeks after EAE induction confirmed that, unlike in PBS- or OVA-pσ1-dosed mice, PLP_139–151_-specific Th1 response was diminished in PLP:OVA-pσ1-dosed mice. Mean + SD of 10–15 mice per group is depicted. * P<0.001 for PLP:OVA-pσ1 vs. PBS. **C.** Mice were dosed with a single 100 µg dose of PLP:OVA-pσ1 or PBS on day +6 relative to the day of EAE induction. Unlike PBS, single dose administration of PLP:OVA-pσ1 significantly inhibited the occurrence and duration of the clinical EAE. * P<0.05 for PLP:OVA-pσ1 vs. PBS. **D.** Mice dosed with PLP:OVA-pσ1 or with PBS 21 days before EAE induction were sacrificed at the peak of the disease (day 14 post-challenge), and histopathology of their spinal cords was determined by staining with luxol fast blue (LFB) and H&E. Mice dosed with PLP:OVA-pσ1 showed significant reduction in the central nervous system (CNS) tissue pathology (designated by arrows) compared to PBS-dosed mice. * P<0.001 for PLP:OVA-pσ1 vs. PBS.

Susceptible female SJL mice nasally dosed with PLP:OVA-pσ1, OVA-pσ1, or PBS were subjected to conventional PLP_139–151_ challenge. PBS- and OVA-pσ1-dosed mice developed EAE with average clinical scores >3 at peak disease, followed by relapsing-remitting disease and never fully recovered (Figure 1A). Mice dosed with PLP:OVA-pσ1 showed delayed development and reduced duration of clinical disease; the average clinical scores at peak disease were ∼1. Unlike PBS- or OVA-pσ1-dosed mice, PLP:OVA-pσ1-protected mice recovered completely from the acute disease following one relapse. PLP_139–151_-specific delayed type hypersensitivity (DTH) responses (Figure 1B) 2 wks after EAE induction confirmed significant reduction of PLP_139–151_-specific Th1 cells by PLP:OVA-pσ1-dosed mice, but not those dosed with OVA-pσ1, revealing the importance of Ag-specificity induced by pσ1-delivered tolerogens.

SJL mice nasally dosed with PLP:OVA-pσ1 prior to EAE induction showed minimal mononuclear cell infiltration with reduced demyelination compared to the PBS-dosed group (Figure 1D; Table 1). FACS analysis performed on spinal cord cells revealed minimal infiltration of inflammatory cells (MHC II^+^CD45^high^) into the central nervous system (CNS), and when compared with the naive controls, only a negligible percentage of Mac-3^+^ macrophages was detectable in the CNS (Table 2). In contrast, PBS-dosed mice showed significant CNS infiltration with CD11b^+^Gr-1^+^ neutrophils, CD4^+^TCR-β^+^ lymphocytes, and Mac-3^+^ macrophages. PLP:OVA-pσ1-mediated protection against EAE is due in part to suppression of encephalitogenic cell infiltration into the CNS.

**Table 1:** Nasal administration of PLP:OVA-pσ1 prior to PLP_139–151_ challenge protects SJL mice from EAE^a^.

Treatment^b^	EAE/Total^c^	Onset^d^	Max.Score^e^	Cs^f^	Inflammation^g^	Demyelination^h^
PBS	12/12	8.83±1.7	5	55.29	2.4±0.5	3.1±0.4
PLP:OVA-pσ1	12/12	11.5±1.1*	2	9.17*	0.5±0.7*	0.7±0.4*

a SJL mice were challenged s.c. with 200 µg PLP_139–151_ in complete Freund's adjuvant plus 200 ng PT i.p. on days 0 and 2.

b Mice were nasally immunized 14 days prior to challenge with 100 µg of PLP:OVA-pσ1 or with PBS.

c Number of mice with EAE/total in group.

d Mean day ± SD of clinical disease onset.

e Maximum (Max.) daily clinical score.

f Cumulative scores (CS) were calculated as the sum of all scores from disease onset to day 26 post-challenge, divided by the number of mice in each group. * P<0.001 for PBS vs. PLP:OVA-pσ1-dosed mice.

g Mean score ± SEM of inflammation: the infiltration of nucleated cells into spinal cords was scored from 0 to 4 in each mouse separately, and the mean score and SEM were calculated. *, P<0.001 for PBS vs. PLP:OVA-pσ1-dosed mice.

h Mean score ± SEM of demyelination: the demyelination in spinal cords was scored from 0 to 4 in each mouse separately, and the mean score and SEM were calculated. *, P<0.001 for PBS vs. PLP:OVA-pσ1-dosed mice.

**Table 2:** Nasal treatment with PLP:OVA-pσ1 after EAE induction^a^ reduce inflammatory cell infiltration^b^ into the spinal cords.

	MHC class II^+^ CD45 ^high^			
Treatment^c^	% Infiltration	% CD4^+^TCR-β^+^	% CD11b^+^Gr-1^+^	% Mac-3^+^
Before EAE	0.94±0.27	0.63±0.06	0.56±0.15	0.03±0.02
PLP:OVA-pσ1	0.83±0.2*	0.52±0.09**	0.24±0.06*	0.32±0.14*
PBS	3.44±0.4	2.78±0.87	2.26±0.18	1.54±0.25

a SJL/J mice were challenged s.c. with 200 µg PLP_139–151_ in complete Freund's adjuvant plus 200 ng PT i.p. on days 0 and 2.

b Results are shown in percentage of MHC class II^+^ CD45^high^ cells from the total cells in spinal cords analyzed by FACS. *, P<0.001 **, P<0.05 for PBS vs. PLP:OVA-pσ1-treated mice.

c Mice were nasally treatment 6 days post-challenge with 100 µg of PLP:OVA-pσ1 or with PBS.

### Single Nasal Dose of PLP:OVA-pσ1 Treats EAE

To investigate the impact of single PLP:OVA-pσ1 dose subsequent EAE challenge, SJL mice were dosed with PLP:OVA-pσ1 or with PBS six days after PLP_139–151_ challenge. All PBS-dosed mice displayed expected disease with the peak average clinical score of 4 (Figure 1C). PLP:OVA-pσ1-treated mice showed delayed onset of EAE and ameliorated clinical disease with the average clinical score of 1 at the peak of disease (Figure 1C), implicating the therapeutic potential of pσ1-delivered auto-Ags.

### PLP:OVA-pσ1 Confers Protection via Stimulation of T_reg_ and FoxP3^+^ CD25^−^CD4^+^ Th2 cells

**Figure 2:**
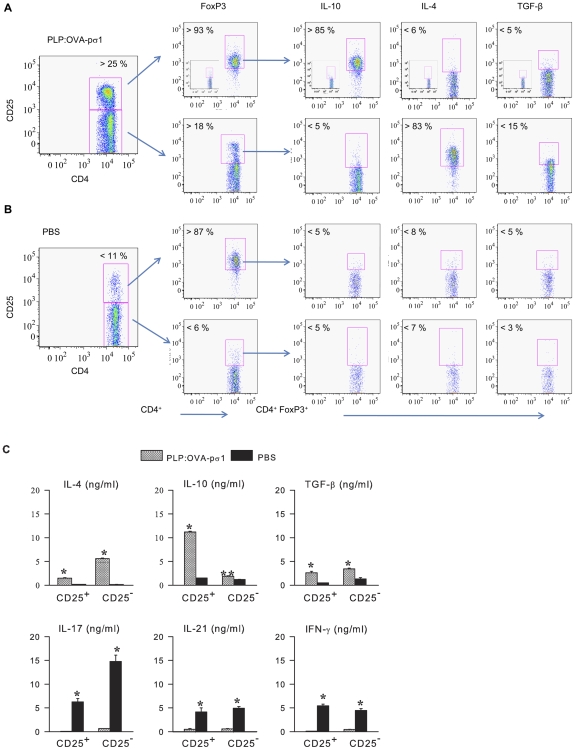
PLP:OVA-pσ1 induces IL-10-producing T_reg_ and IL-4-producing FoxP3^+^Th2 cells. Mice were dosed with 100 µg of PLP:OVA-pσ1 **A** and **C** or with PBS **B** and **C** fourteen days before challenge with PLP_139–151_ peptide and sacrificed 2 weeks later. **A** and **B.** Lymphocytes from head and neck LNs (HNLNs) and spleens were isolated, cultured with 30 µg/ml of PLP_139–151_ for 3 days, and stained for expression of extracellular (CD25, CD4 and TGF-β), and intracellular markers (FoxP3, IL-10, and IL-4). Presented FACS plots show cells isolated from spleens with respective isotype controls provided in inserts. Unlike PBS-dosed mice, PLP:OVA-pσ1 administration induced significant enrichment in FoxP3^+^ T_reg_ cells and CD25^−^ Th2 cells in mice. In contrast to PBS-derived CD4^+^ T cells, FoxP3^+^ T_reg_ cells from PLP:OVA-pσ1-dosed mice produced predominantly IL-10, whereas PLP:OVA-pσ1-derived FoxP3^+^ Th2 cells produced IL-4. Average percentage of 10 mice/group is depicted. * P<0.05 for PLP:OVA-pσ1 vs. PBS. **C.** CD25^+^CD4^+^ and CD25^−^CD4^+^ T cells were bead-sorted from combined HNLN, MLN, and splenic lymphocytes and in vitro stimulated with plate bound anti-CD3 and soluble anti-CD28 mAbs for 72 h. Collected supernatants were analyzed by cytokine ELISA. Negligible amounts of proinflammatory cytokines were secreted by CD4^+^ T cells isolated from PLP:OVA-pσ1-dosed and challenged mice. Mean ± SEM of 10 mice/group is depicted. * P<0.05 for PLP:OVA-pσ1 vs. PBS.

CD4^+^ T cells isolated from PLP:OVA-pσ1- and PBS-dosed mice after EAE challenge were evaluated by flow cytometry to determine their T_reg_ cell composition. Nasal PLP:OVA-pσ1-dosed mice showed a significant augmentation in FoxP3^+^ CD25^+^CD4^+^ T cells and FoxP3^+^ CD25^−^CD4^+^ Th2 cells, when compared to PBS-dosed mice (Figure 2). PLP:OVA-pσ1 induced >25% of CD4^+^CD25^+^ T cells, of which >93% were FoxP3^+^ and CD25^−^CD4^+^ T cells of which >18% were FoxP3^+^ (Figure 2A; Table S1), unlike PBS-dosed mice showing only a slight enrichment in T_reg_ cells (∼10%) and CD25^−^ Th2 cells as naive mice (Figure 2B). CD4^+^ T cells from PLP:OVA-pσ1-dosed mice showed >85% T_reg_ cells expressing IL-10 and nearly as many CD25^−^ Th2 cells producing IL-4 and significantly more TGF-β than those from PBS-dosed mice. Proinflammatory cytokines, IL-17, IL-21, and IFN-γ, were produced primarily by PBS-derived CD4^+^ T cells and only nominally by those from PLP:OVA-pσ1-dosed mice (Figure 2C). Thus, nasal administration of PLP:OVA-pσ1 induced Ag-specific, anti-inflammatory T_reg_ and Th2 cells.

### IL-10R Blockade Abolishes PLP:OVA-pσ1-Derived T_reg_ Cells' Protective Efficacy

**Figure 3:**
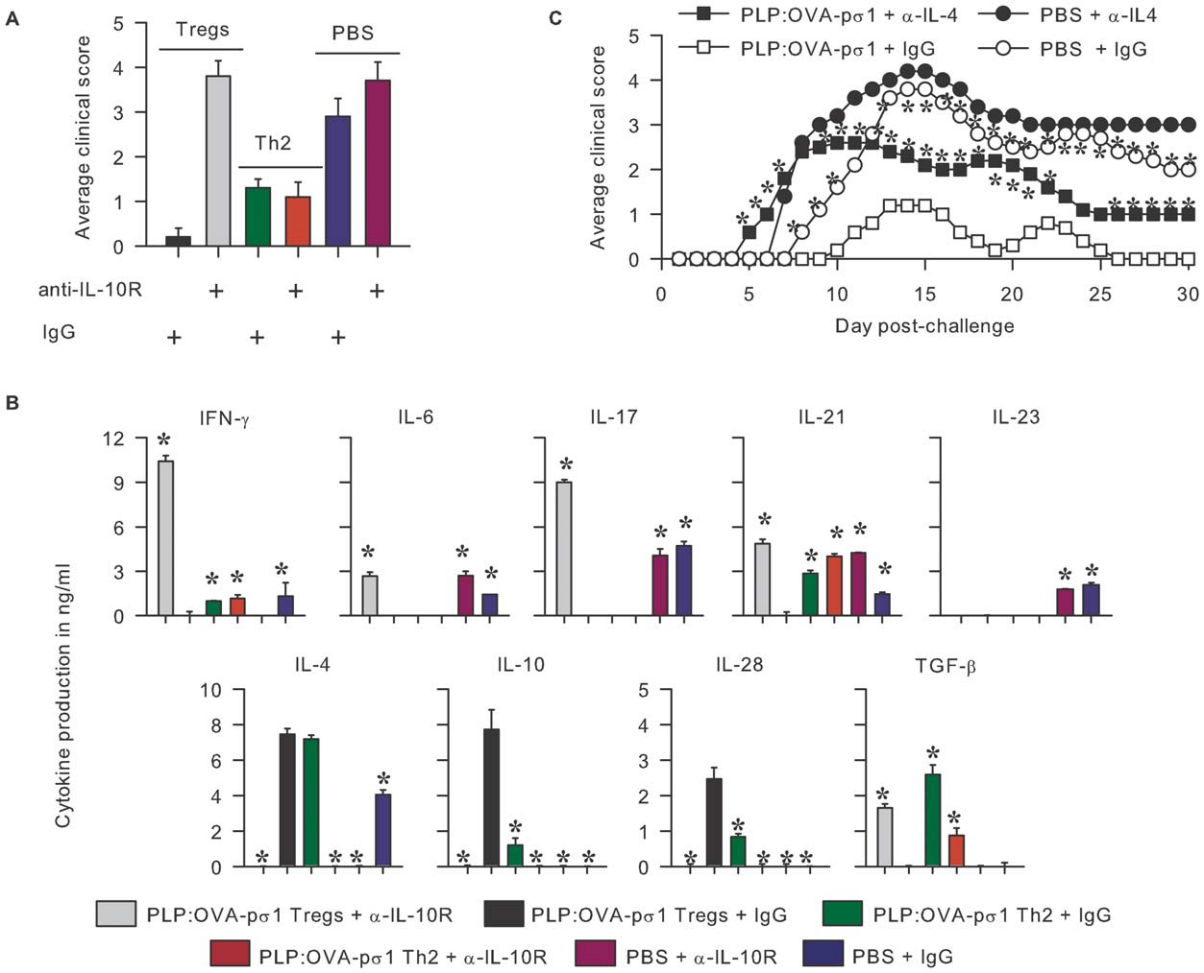
PLP:OVA-pσ1 protects against EAE via IL-10-producing T_reg_ and IL-4-producing Th2 cells. Mice were dosed with PLP:OVA-pσ1, and two weeks later, T_reg_ and CD25^−^CD4^+^ Th2 cells were isolated and adoptively transferred to naive recipients. On day 0, mice were induced with EAE and on days −1 and +5 received anti-IL-10R mAb or IgG isotype control Ab. **A.** In contrast to T_reg_ cells + IgG-treated mice, mice given T_reg_ cells + anti-IL-10R mAb developed clinical EAE. Administration of anti-IL-10R mAb had no effect on the clinical disease in mice adoptively transferred with PLP:OVA-pσ1-derived Th2 cells. * P<0.05 vs. PBS + IgG. **B.** Mice were sacrificed at the peak of clinical disease, and CD4^+^ T cells isolated from their LNs were evaluated for production of cytokines by ELISA. T_reg_ cells + anti-IL-10R-treated mice produced significantly more proinflammatory cytokines and less anti-inflammatory cytokines when compared to T_reg_ cells + IgG-treated mice. Mean and SD of 5 mice per group is depicted; * P<0.05 vs. T_reg_ cells + IgG. **C.** Mice dosed with PLP:OVA-pσ1 or PBS on days −14 and −7 were injected with anti-IL-4 mAb or rat IgG on days −1 and +5. PBS + anti-IL-4-dosed mice developed accelerated and more severe EAE than PBS + IgG-dosed mice. The disease in PLP:OVA-pσ1 + anti-IL-4-dosed mice was less severe than in PBS + IgG-dosed mice, but significantly more severe than in PLP:OVA-pσ1 + IgG-dosed mice. * P<0.05 for PLP:OVA-pσ1 + IgG vs. PBS + IgG or PLP:OVA-pσ1 + anti-IL-4.

To investigate the relative contribution to protection by these PLP-specific T_reg_ and Th2 cells, naive SJL mice were adoptively transferred with PLP:OVA-pσ1-derived T_reg_ or Th2 cells and treated with anti-IL-10R mAb 1 day prior and 5 days after EAE induction. Adoptive transfer of Ag-specific T_reg_ cells nearly abrogated EAE, whereas, Th2 cells partially ameliorated disease (Figure 3A; Figure S1). Anti-IL-10R mAb treatment had no effect upon PLP:OVA-pσ1-derived Th2 cells to prevent EAE; however, IL-10R blockade in recipients given PLP:OVA-pσ1-derived T_reg_ cells rendered them susceptible to EAE (Figure 3A).

Such pronounced clinical disease by anti-IL-10R-treated T_reg_ cell recipients showed enhanced proinflammatory responses by >11- and 9-fold increases in IFN-γ and IL-17, respectively, by LN CD4^+^ T cells when compared to IgG-treated T_reg_ cell recipients (Figure 3B). Likewise, IL-6 and IL-21 were augmented 3- and 5-fold, respectively, when compared to lymphocytes from T_reg_ cell recipients treated with IgG. Consequently, T_reg_ cells+anti-IL-10R mAb-treated recipients did not produce IL-4, IL-10, or IL-28, but did produce ∼2-fold more TGF-β when compared to T_reg_ cell recipients treated with IgG. Lymphocytes from IgG-treated T_reg_ cell recipients resembled PLP:OVA-pσ1-treated mice, producing anti-inflammatory cytokines, IL-4, IL-10, and IL-28, and suppressing TGF-β production. Diseased mice from groups treated with PBS + IgG or PBS + anti-IL-10R mAb showed pronounced inflammatory responses evident by augmented IL-6, IL-21, and IL-23, in addition to >5-fold increases in IL-17 when compared to IgG-treated T_reg_ cell recipients (Figure 3B). IgG-treated Th2 cell recipients produced significantly more IFN-γ, at least 3-fold more IL-21 and TGF-β, considerably less IL-10 and IL-28, but similar amounts of IL-4 when compared to IgG-treated T_reg_ cell recipients. Lymphocytes from anti-IL-10R-treated Th2 cell recipients showed inhibition of IL-4, IL-28, and 3-fold less TGF-β when compared to IgG-treated Th2 cell recipients, and yet both groups showed reduced EAE because of their ability to inhibit IFN-γ, IL-6, and IL-17.

### IL-4 Neutralization Partially Reverses PLP:OVA-pσ1-Mediated Protection

Testing the relevance of IL-4 in PLP:OVA-pσ1-mediated protection against EAE, groups of mice were dosed with PLP:OVA-pσ1 or with PBS on days -14 and -7, subsequently treating them with an anti-IL-4 mAb or rat IgG on days −1 and +5 relative to EAE challenge. Mice dosed with PBS+IgG developed typical EAE onset (Figure 3C). IL-4 neutralization accelerated onset of clinical disease and amplified disease severity in PBS-dosed mice (Figure 3C; Table S2). PLP:OVA-pσ1 + anti-IL-4 mAb-treated mice also showed earlier disease onset and greater EAE severity than in PLP:OVA-pσ1 + IgG-dosed mice, but were less pronounced than in PBS + IgG-dosed control mice.

CD4^+^ T cells isolated from anti-IL-4 mAb-treated and PLP:OVA-pσ1-dosed mice at the peak of the disease showed pronounced proinflammatory cytokines, IFN-γ, IL-6, and IL-17, and reduced IL-10 when compared to PLP:OVA-pσ1 + IgG-dosed mice (Figure S2). Overall, IL-4 neutralization in either PBS- or PLP:OVA-pσ1-dosed mice induced more IFN-γ, when compared to their respective IgG-treated control mice, and significantly less IL-10 was produced in anti-IL-4 mAb-treated PLP:OVA-pσ1-dosed mice than in PLP:OVA-pσ1 + IgG-protected mice.

### Functional Inactivation of CD25^+^ T Cells Abrogates PLP:OVA-pσ1-Mediated Tolerance and Stimulates TGF-β-Dependent EAE

**Figure 4:**
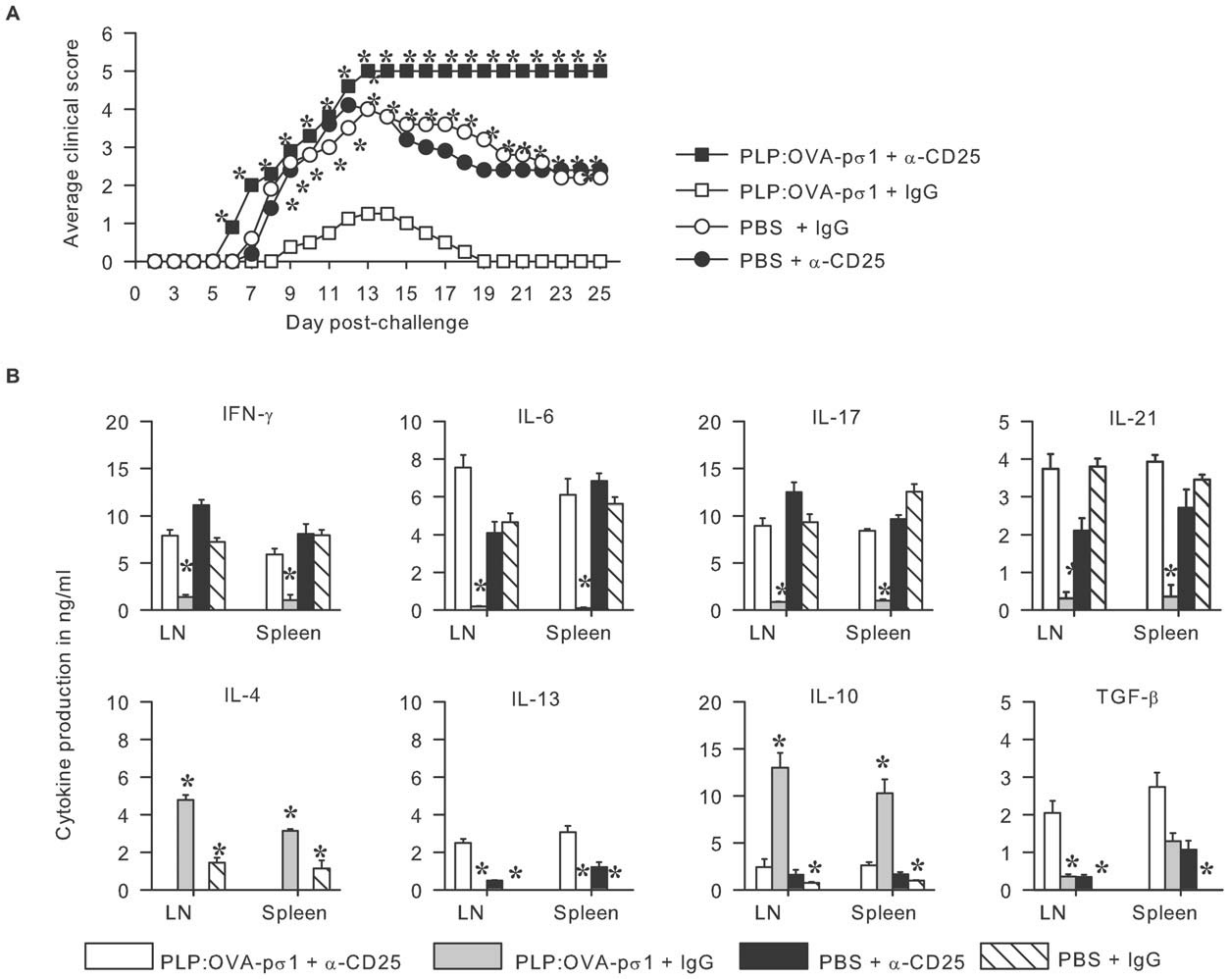
T_reg_ cells are important for PLP:OVA-pσ1-induced protection against EAE. Mice dosed with PLP:OVA-pσ1 or PBS on days −14 and −7 were treated with anti-CD25 mAb or rat IgG on days −5 and −2. **A.** Clinical disease in PBS-dosed mice was not affected by anti-CD25 mAb. PLP:OVA-pσ1 + anti-CD25-treated mice developed severe EAE, and all succumbed to the disease. Averaged clinical scores for 10 mice per group are shown. * P<0.05 for PLP:OVA-pσ1 + anti-CD25- and PBS + IgG- vs. PLP:OVA-pσ1 + IgG-treated mice. **B.** CD4^+^ T cells were cultured with feeder cells and PLP_139–151_ peptide for 72 h. Functional inactivation of T_reg_ cells in PLP:OVA-pσ1-dosed mice resulted in induction of proinflammatory CD4^+^ T cells, producing IFN-γ, IL-6, IL-17, IL-21, and TGF-β. Importantly, CD4^+^ T cells obtained from PLP:OVA-pσ1-dosed anti-CD25 mAb-treated mice produced more IL-13 in LNs and spleens and more TGF-β in LNs than in any other experimental group. Mean + SEM from 5 mice per group is shown. *, P<0.05 for PLP:OVA-pσ1 + anti-CD25 vs. PLP:OVA-pσ1 + IgG and PBS + IgG.

Adoptive transfer of T_reg_ cells from PLP:OVA-pσ1-primed mice was completely protective against EAE challenge (Figures 3A and S1), further implicating relevance of T_reg_ cells for protection. Neutralization of functional T_reg_ cells did not affect course of EAE in PBS-dosed mice when compared to PBS + IgG-dosed mice (Figure 4A); however, EAE accelerated in anti-CD25 mAb-treated PLP:OVA-pσ1-dosed mice, resulting in a notably more severe EAE (Figure 4A). The protective regulatory and Th2-type responses normally induced by PLP:OVA-pσ1 were abated subsequent CD25 neutralization. The impact was evident by 3-fold reductions in IL-10, loss of IL-4, and augmented proinflammatory responses noted by 5-fold for IFN-γ, 8-fold for IL-17, and >10-fold enhancements for IL-6 and IL-21, as well as increased TGF-β (Figure 4B). PBS-dosed mice treated with anti-CD25 mAb or with IgG developed classic proinflammatory responses, and LN CD4^+^ T cells isolated from these mice showed elevations in IFN-γ, IL-6, IL-17, and IL-21 and considerably less IL-10 and IL-4 when compared to PLP:OVA-pσ1 + IgG-dosed mice (Figure 4B). PLP:OVA-pσ1 + IgG-treated mice produced the expected elevations in IL-4 and IL-10 and near neutralization of proinflammatory cytokines in a PLP_139–151_-specific T_reg_ cell-dependent fashion. Moreover, the lack of protection in CD25-neutralized PLP:OVA-pσ1-dosed mice was associated with enhanced proinflammatory cytokines and a striking induction of TGF-β.

### TGF-β and CD25 Co-Neutralization Restores pσ1-Mediated Protection against EAE Independent of T_reg_ Cells

**Figure 5:**
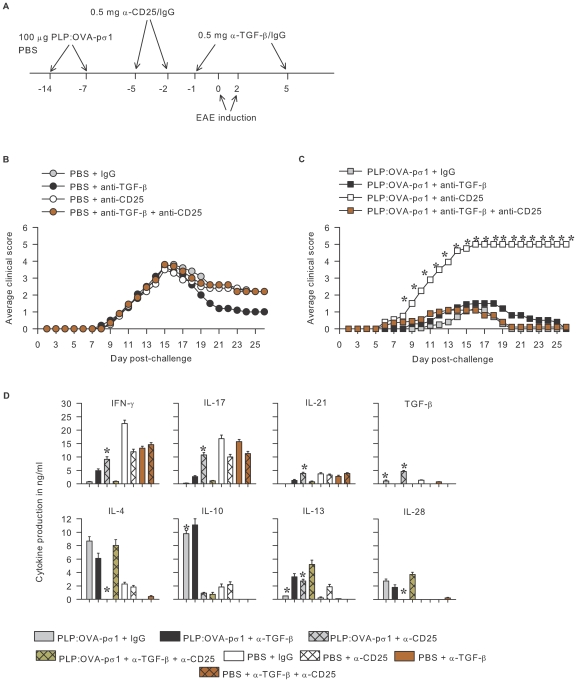
Abated protection against EAE in anti-CD25 mAb-treated PLP:OVA-pσ1-dosed mice is restored upon TGF-β co-neutralization. **A.** Experimental design for neutralization of TGF-β and CD25. **B.** PBS-dosed mice, independent of treatment, developed expected course of EAE. **C.** Treatment with anti-CD25 mAb abrogated PLP:OVA-pσ1-mediated protection against EAE, but concomitant treatment with anti-TGF-β and anti-CD25 mAbs restored PLP:OVA-pσ1-induced protection. Mean of 10 mice per group is shown. **B** and **C.** * P<0.05 vs. IgG-dosed mice. **D.** HNLN CD4^+^ T cells on day 10 post-EAE induction were cultured with PLP_139–151_ peptide for 72 h. PBS-dosed mice independent of Ab treatment produced elevated proinflammatory cytokines IFN-γ, IL-17, and IL-21, and little to no IL-4, IL-10, IL-13, and TGF-β. However, levels of IFN-γ and IL-17 produced by CD4^+^ T cells were diminished in PBS + anti-TGF-β-treated mice when compared to PBS + IgG- treated mice. PLP:OVA-pσ1-protected mice treated with IgG, anti-TGF-β mAb, or anti-TGF-β + anti-CD25 mAbs produced enhanced IL-4 and IL-28, but minimal proinflammatory cytokines. Additionally, mice dosed with PLP:OVA-pσ1 + anti-CD25 + anti-TGF-β mAbs produced significantly more IL-13 and IL-4 than diseased PLP:OVA-pσ1 + anti-CD25-dosed mice. Mean + SEM of 5 mice per group is shown * P<0.05 for the PLP:OVA-pσ1 + anti-TGF-β + anti-CD25 vs. PLP:OVA-pσ1 + IgG and PLP:OVA-pσ1 + anti-CD25.

T_reg_ cell neutralization exacerbated EAE, negating the protective capacity of PLP:OVA-pσ1, resulting in enhanced TGF-β production (Figure 4B) and implicating a proinflammatory role of this cytokine in EAE. To address TGF-β's participation in EAE development subsequent CD25 neutralization, PLP:OVA-pσ1- and PBS-dosed mice were treated in vivo with anti-TGF-β mAb, anti-CD25 mAb, both, or IgG (Figure 5A). No difference in onset or disease severity was observed between mice dosed with PBS and treated with the different combinations of mAbs, except those mice dosed with PBS + anti-TGF-β mAb recovered sooner from the acute disease (Figure 5B). In contrast to PLP:OVA-pσ1 + anti-CD25 mAb-treated mice, which developed a very aggressive disease, mice given PLP:OVA-pσ1 + any of the remaining Ab treatments developed only very mild disease with the average peak clinical score of ∼1.5; all of these mice recovered from acute EAE (Figure 5C). Thus, co-neutralization of CD25 and TGF-β in PLP:OVA-pσ1-treated mice resembled ameliorated disease, as seen in PLP:OVA-pσ1 + IgG-dosed mice. These results showed that mice functionally neutralized of their CD25^+^ T_reg_ cells in the presence of tolerogen develop a more aggressive, TGF-β-dependent EAE. The suppressive activity of PLP:OVA-pσ1 could only be restored upon co-neutralization of TGF-β. These findings corroborate the results in Figure 3B in which minimal to no TGF-β was detected in PBS-treated mice, suggesting that TGF-β has a minimal role in PLP-mediated EAE.

### Restoration of Tolerance by PLP:OVA-pσ1 upon Co-Neutralization of CD25 and TGF-β Is IL-28-Dependent

Examination of cytokine profiles conducted 10 days post-EAE challenge revealed that PLP_139–151_-restimulated mononuclear cells from PBS-dosed groups produced elevated amounts of IL-6, IL-17, IL-21, and IL-23, and very little to no anti-inflammatory IL-4 and IL-10 (Figure S3). When compared to PLP:OVA-pσ1 + IgG-treated mice, PLP:OVA-pσ1 + anti-CD25 mAb + anti-TGF-β mAb-treated mice showed a slight reduction in IL-4, but a marked 7.3-fold reduction in IL-10, which was consistent with the lack of functional IL-10-producing T_reg_ cells. In addition, this treatment also reduced IL-22 by 7.7-fold and showed inhibition of IL-6, IL-17, IL-21, and IL-23 (Figure S3). IL-28 production was preserved by this treatment; however, when compared to diseased PLP:OVA-pσ1 + anti-CD25 mAb-treated mice, IL-28 was enhanced 16.4-fold, but with no change in IL-10, implicating that in the absence of functional T_reg_ cells, PLP:OVA-pσ1-induced protection can occur via Th2-type cells. IL-28 was not produced in diseased mice treated with either PBS plus the various mAbs, or with PLP:OVA-pσ1 + anti-CD25 mAb, showing the anti-inflammatory property of IL-28 in EAE. Lack of differences in IL-4-secretion between mice dosed with PLP:OVA-pσ1 + IgG or PLP:OVA-pσ1 + anti-TGF-β + anti-CD25 mAb further suggests that the presence of T_reg_ cells is not required for IL-4 production (Figure 5D; Figure S3).

Although T_reg_ cells were absent in mice treated with anti-CD25 plus anti-TGF-β mAbs, upon tolerance induction with PLP:OVA-pσ1, alternative regulatory T cells were induced evident by the expression of FoxP3 by CD25^−^ CD4^+^ T cells (Table 3). In fact, the inclusion of anti-TGF-β mAb in the treatment paradigm restored elevated FoxP3 expression by CD25^−^CD4^+^ T cells to levels similar to those obtained from PLP:OVA-pσ1 + IgG-dosed mice. In contrast, PLP:OVA-pσ1 + anti-CD25 mAb-treated mice displayed a 67% reduction in FoxP3^+^CD25^−^CD4^+^ T cells when compared to PLP:OVA-pσ1-protected mice (Table 3). PLP:OVA-pσ1 + anti-TGF-β mAb treatment showed only a 20% reduction in FoxP3^+^CD25^−^CD4^+^ T cells; these mice still retained their T_reg_ cells.

**Table 3:** In vivo neutralization of TGF-β and CD25 induces FoxP3 expression of PLP:OVA-pσ1-primed Th2 cells.

Treatment^a^	% FoxP3^+^CD25^−^CD4^+b^
PLP:OVA-pσ1 + IgG	24.78±0.96^+,§^
PLP:OVA-pσ1 + anti-TGF-β	19.73±1.31**
PLP:OVA-pσ1 + anti-CD25	8.12±1.16
PLP:OVA-pσ1 + anti-TGF-β + anti-CD25	26.68±0.51*,^§^

a SJL/J mice were challenged s.c. with 200 µg PLP_139–151_ in complete Freund's adjuvant plus 200 ng PT i.p. on days 0 and 2, and were nasally dosed 14 and 7 days prior to EAE challenge with 100 µg of PLP:OVA-pσ1 or with PBS and treated as described in Fig. 5.

b Average percentages ± SD of FoxP3^+^CD25^−^CD4^+^ T cells as a fraction of CD4^+^ T cells from 3 mice per group are depicted, *, P<0.001, **, P<0.05 vs. PLP:OVA-pσ1 + anti-CD25, ^§^, P<0.05 vs. PLP:OVA-pσ1 + anti-TGF-β ~.

**Figure 6:**
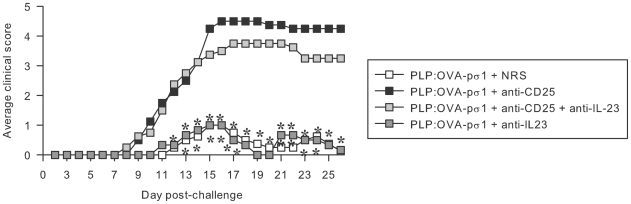
IL-23-independent induction of Th17 cells in T_reg_ cell-depleted PLP:OVA-pσ1-dosed mice. SJL mice were nasally dosed with PLP:OVA-pσ1 or PBS on days −14 and −7 relative to EAE induction. Mice were i.p. injected with anti-CD25/rat IgG and anti-IL-23p19 rabbit serum (RS) or normal rabbit serum (NRS). Neutralization of IL-23 did not suppress clinical disease in T_reg_ cell-inactivated PLP:OVA-pσ1-dosed mice. Mean of 5 mice per group is shown. * P<0.05 vs. PLP:OVA-pσ1 + anti-CD25-treated mice.

Aside from TGF-β, IL-23 also has been shown to activate Th17 cells [27]–[29]. To determine the necessity of IL-23 for induction of EAE in PLP:OVA-pσ1 + anti-CD25 mAb-treated mice, mice were co-treated with anti-IL-23p19 serum. Neutralization of IL-23 had no effect on clinical onset and severity of EAE (Figure 6).

### Endogenous IL-28 Protects against EAE in the Absence of T_reg_ Cells

**Figure 7:**
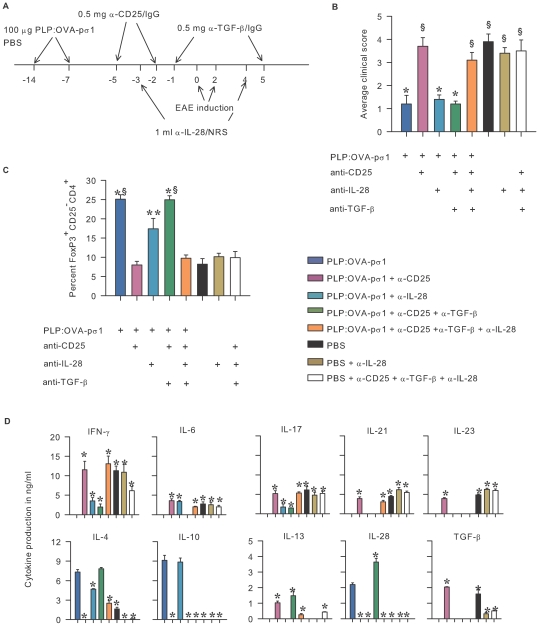
IL-28 is responsible for protection against EAE in T_reg_ cell-depleted PLP:OVA-pσ1-dosed mice. **A.** Mice were nasally dosed with 100 µg of PLP:OVA-pσ1 or with PBS on days −14 and −7 relative to EAE induction (day 0). Mice were treated with anti-CD25 mAb on days −5, and −2; anti-TGF-β mAb on days −1 and +5, and/or with anti-IL-28 RS on days −3 and +4, relative to EAE induction. **B.** In contrast to PLP:OVA-pσ1 (+ NRS)- or PLP:OVA-pσ1 + anti-IL-28 RS-treated mice, mice treated with PLP:OVA-pσ1 + anti-CD25 mAb or PLP:OVA-pσ1 + anti-CD25 mAb + anti-TGF-β mAb + anti-IL-28 RS developed pronounced EAE. **C.** Pooled lymphocytes from MLNs and HNLNs at the peak of EAE were stained for FACS and analyzed for expression of FoxP3. In contrast to PLP:OVA-pσ1-protected mice that received NRS, anti-IL-28 RS, or combination of anti-CD25 and anti-TGF-β mAbs, mice that received PLP:OVA-pσ1 + anti-CD25 mAb + anti-TGF-β mAb + anti-IL-28 RS or PLP:OVA-pσ1 + anti-CD25 mAb showed significantly reduced percentages of FoxP3^+^ Th2 cells. **B** and **C.** * P<0.001, ** P<0.05 vs. PLP:OVA-pσ1 + anti-CD25, ^§^ P<0.05 vs. PLP:OVA-pσ1 + anti-IL-28. **D.** Pooled HNLN and MLN CD4^+^ T cells were evaluated for production of proinflammatory and anti-inflammatory cytokines by ELISA. CD4^+^ T cells from mice treated with PLP:OVA-pσ1 + anti-IL-28 RS produced similar amounts of anti-inflammatory cytokines and slightly elevated amounts of proinflammatory cytokines when compared to PLP:OVA-pσ1 (+ NRS)-dosed mice. In contrast to PLP:OVA-pσ1-dosed mice, CD4^+^ T cells from mice dosed with PLP:OVA-pσ1 + anti-CD25 + anti-TGF-β mAbs + anti-IL-28 RS displayed cytokine profile that closely resembled PLP:OVA-pσ1 + anti-CD25-treated mice. Mean and SD of 5 mice per group are depicted; * P<0.05 vs. PLP:OVA-pσ1-dosed mice.

To investigate the role of IL-28 in the PLP:OVA-pσ1-mediated protection against EAE in the absence of functional T_reg_ cells, mice dosed with PLP:OVA-pσ1 or PBS were treated with anti-IL-28 rabbit serum (RS), normal RS (NRS), and/or with mAbs to CD25 and TGF-β (Figure 7A). Treatment of PBS- or PLP:OVA-pσ1-dosed mice with anti-IL-28 RS did not significantly alter EAE when compared to their respective control groups dosed with NRS (Figure 7B). Neutralization of IL-28 in concert with co-neutralized CD25 and TGF-β rendered PLP:OVA-pσ1-dosed mice susceptible to EAE development, presumably due to the significant reduction in FoxP3^+^ Th2 cells (Figure 7C), reaffirming the importance of IL-28-mediated protection in EAE.

As before, CD4^+^ T cells from PLP:OVA-pσ1 + NRS- and PLP:OVA-pσ1 + anti-CD25 + anti-TGF-β-treated mice produced predominantly anti-inflammatory cytokines (Figure 7D). Although PLP:OVA-pσ1 + anti-IL-28-dosed mice conferred protection against EAE (Figure 7B), upon peptide restimulation, their CD4^+^ T cells produced proinflammatory cytokines, IFN-γ, IL-6, and IL-17 (Figure 7D), and these mice expressed fewer FoxP3^+^ Th2 cells (Figure 7C), unlike PLP:OVA-pσ1 + NRS-treated mice that were protected against EAE, but without inducing proinflammatory cytokines. Such differences in protection were not attributed to IL-10 since these remained unchanged in both groups. Neutralization of IL-28 in PBS or PBS + anti-CD25 + anti-TGF-β-dosed mice did not significantly affect proinflammatory cytokine production by CD4^+^ T cells; however, secretion of IL-4 and TGF-β was significantly reduced in these mice when compared to PBS + NRS-dosed mice. Independent of treatment, all PBS-dosed mice showed only marginal production of anti-inflammatory cytokines. These studies demonstrate IL-28 can be induced to supplant protective IL-10^+^ T_reg_ cells. To our knowledge, this is the first such report describing IL-28's protective capacity against inflammatory autoimmune diseases.

## Discussion

Tolerance is the active inability to respond to self or a defined Ag and represents a method to abolish self-reactivity to ultimately enable protection against autoimmune disease. A caveat in preventing or treating autoimmune diseases in humans is being able to successfully adapt tolerance methods used in various animal models. In a double-blind phase III clinical trial to test the feasibility of inducing oral tolerance to myelin basic protein and PLP, high doses of myelin Ags were administered, and although TGF-β-secreting CD4^+^ T cells were induced [30], no differences in MS outcomes between placebo and treated were observed [31]. Notwithstanding, alternative methods of mucosal delivery, particularly sublingual, have proven effective in humans to induce tolerance to alleviate allergies to dust mites and grass pollens [32]–[34]. Rendering tolerance via sublingual delivery of these allergens requires contact with the oral mucosa since immediate swallowing of allergens diminishes the tolerogenic capacity [33]. To a limited extent, targeting auto-Ags to the mucosal epithelium has been tested using cholera toxin subunit B (CT-B) to improve diabetes [35] and EAE [36] in mice. However, CT-B also behaves as a mucosal adjuvant [37], [38], resulting in conflicting outcomes when Ags chemically coupled to CT-B produce potent Ag-specific immunity [36], [39] or induce tolerance [35], [40]. Since chemical modification can render a tolerogen immunogenic, the alternative approach of genetically fusing PLP_139–151_ to CT-B maintains its ability to remain tolerogenic and suppress EAE [24]. Despite its success, this approach again requires multiple nasal administrations to maintain efficacy against EAE. These collective studies implicate the potential of induced tolerance if given by a suitable mucosal route, allowing for tolerogen uptake. Thus, there have been limited attempts to test the potential of microbial adhesins in humans to facilitate tolerance induction, let alone, with pσ1.

To enable this latter possibility and to take advantage of pσ1's adhesive properties [5], [41], OVA-pσ1 was modified with two copies of immunodominant T cell epitope PLP_130–151_, resulting in a functional PLP:OVA-pσ1, which, when given nasally, protected susceptible SJL mice against EAE. PLP:OVA-pσ1 protection resulted in the diminution of proinflammatory CD4^+^ T cells and the stimulation of CD4^+^ T cells producing regulatory and anti-inflammatory cytokines. The related protein lacking PLP peptide, OVA-pσ1, was not protective, further showing the tolerogenic responses being Ag-specific. Importantly, the therapeutic effect conferred by PLP:OVA-pσ1 could be rendered with as low as a single 100 µg dose, similar to that shown for OVA-specific tolerance [5]. While tolerance to encephalitogenic proteins or peptides when applied mucosally can be shown, these generally require multiple and/or large Ag doses to confer protection against EAE [3], [17], [19], [20], [42].

Although PLP:OVA-pσ1 is a derivative of OVA-pσ1, this was done to facilitate monitoring of B cell responses subsequent mucosal delivery of this tolerogen since the PLP_139–151_ T cell epitope produced weak to no Ab responses in PBS-treated, PLP_139–151_-challenged mice (data not shown). The intent of this work is to establish the potential of pσ1-based therapeutics for MS, and subsequent work will fashion a pσ1-based fusion tolerogen bearing relevant human T and B cell epitopes for MS.

PLP:OVA-pσ1-mediated protection was facilitated by IL-10-producing FoxP3^+^ T_reg_ cells and supported by IL-4-producing FoxP3^+^CD25^−^CD4^+^ Th2 cells. IL-10R blockade abolished protection mediated by PLP:OVA-pσ1-derived T_reg_ cells, allowing for uninhibited Th1- and Th17-type cytokine production with concomitant reductions in IL-4 and IL-28, resulting in attrition to EAE. Such findings regarding IL-10's importance in protection against EAE are similar with what others have shown [2], [4], [17], [43], [44]. Likewise, pσ1-mediated tolerance could not be established in IL-10^−/−^ mice presumably due to the failure in the generation of FoxP3^+^ T_reg_ cells [5].

IL-4 neutralization only partially altered PLP:OVA-pσ1-mediated protection against EAE evident as reduced IL-10 production with concomitant stimulation of proinflammatory Th1- and Th17- type cytokines. Investigating their relative contributions, adoptive transfer of PLP:OVA-pσ1-primed T_reg_ cells prior to PLP_139–151_ peptide challenge conferred complete protection against EAE, but transfer of PLP:OVA-pσ1-primed CD25^−^CD4^+^ Th2 cells, while not conferring complete protection, significantly delayed the disease onset and reduced disease severity. In agreement with our previous study in which OVA-pσ1-mediated tolerance was supported by IL-4-producing Th2 cells, nasal PLP:OVA-pσ1 induced significant increases in FoxP3^+^CD25^−^CD4^+^ Th2 cells, >80% of which produced IL-4. The anti-inflammatory potential of IL-4 has been well characterized [1], [45], and stimulation of regulatory FoxP3^+^CD25^−^CD4^+^ T cells has been shown by us and others [4], [5], [7], [9]. Alternatively, it is plausible that PLP:OVA-pσ1-induced FoxP3^+^ Th2 cells are undergoing conversion to the T_reg_ cells, since conversion of FoxP3^+^CD25^−^CD4^+^ T cells potently inhibited CD4^+^ T cells' expansion in vivo and proliferation in vitro [9], [46]. It is reported that IL-4 can supplement suppressive function of TGF-β-secreting regulatory Th3 cells [20]. IL-4 has also been implicated in triggering expression of FoxP3 on naive peripheral CD25^−^CD4^+^ T cells [47], and when secreted by Th2 cells, IL-4 supports the inhibition of PLP_139–151_-specific proinflammatory responses [7]. Surprisingly, IL-4 deficiency has not been associated with increased susceptibility to autoimmune diseases, suggesting a supportive role for IL-4 in suppression of inflammatory responses against self-Ags [48]. Therefore, consistent with the supportive role of IL-4 in induction of T_reg_ cells [47], possibly IL-4 neutralization downregulates IL-10-producing T_reg_ cells in PLP:OVA-pσ1-dosed mice.

Inquiry into the role of Th2 cytokines to pσ1-dependent tolerance found that the functional inactivation of T_reg_ cells greatly impaired the PLP:OVA-pσ1-induced protection against EAE. Anti-CD25 mAb treatment relinquished the tolerogenic property of PLP:OVA-pσ1, making it more immunostimulatory, as evident by the increased disease severity and enhanced production of TGF-β, IL-6, IL-17, IL-21, and IFN-γ with concomitant reductions in IL-4, IL-10, and IL-28. Although IL-13 was enhanced, our preliminary studies implicated lack of proinflammatory function for this cytokine in T_reg_ cell-depleted PLP:OVA-pσ1-dosed mice. In contrast to concomitant inhibition of CD25 and IL-23, simultaneous neutralization of TGF-β and CD25 reestablished PLP:OVA-pσ1-induced protection, suggesting that anti-CD25 mAb-induced Th17 inflammatory responses in PLP:OVA-pσ1-dosed mice are mediated via TGF-β rather than IL-23. In addition to its established proinflammatory role [28], [29], TGF-β can be a major regulatory cytokine secreted by T_reg_ cells, and its production has been linked to potent suppression of EAE [3], [7].

Protected PLP:OVA-pσ1 + anti-TGF-β-treated mice showed a modest, although significant, decrease in FoxP3^+^ Th2 cells when compared to PLP:OVA-pσ1 + IgG-dosed mice, but these cells remained significantly enhanced when compared to PLP:OVA-pσ1 + anti-CD25-treated diseased mice. Although TGF-β can support conversion of CD25^−^CD4^+^ T cells to T_reg_ cells via enhanced FoxP3 expression [9], [49], our data showed that CD25 neutralization upon PLP:OVA-pσ1 treatment results in enhanced TGF-β responses reminiscent of TGF-β's inflammatory properties, perhaps because of the increased presence of IL-6 [29] and/or IL-21 [50].

Mice co-neutralized of the T_reg_ cells and TGF-β, but dosed with PLP:OVA-pσ1, reestablished tolerance evidenced by the inhibition of Th1 and Th17 cells and the enhanced production of IL-4, IL-13, and IL-28. Reversion of IL-10 production was not evident presumably because of neutralization of T_reg_ cells. Concomitant neutralization of TGF-β, CD25, and IL-28 reversed the Th2 cell-dependent tolerance by PLP:OVA-pσ1 treatment, resulting in EAE and reaffirming the novel role for IL-28 in protection against EAE in the absence of T_reg_ cells. Combined with their enhanced expression of FoxP3, these results suggest an alternative regulatory pathway that can be induced by PLP:OVA-pσ1, but independent of conventional T_reg_ cells. In some cases, minimal levels of IL-10 were still being secreted by CD4^+^ T cells in PLP:OVA-pσ1 + anti-CD25 + anti-TGF-β-dosed mice. Consequently, the possibility of potential synergistic or priming effect by IL-10 upon IL-28 cannot be excluded to account for the observed protection against EAE in the absence of functional T_reg_ cells and proinflammatory TGF-β. A growing body of evidence suggests that type I and II IFNs can induce proinflammatory potential of IL-10 by switching the balance of IL-10 STAT activation from Stat3 to Stat1 [51], [52]. Type III IFNs share functional and structural similarities with type I IFNs, including the Jak-STAT signaling pathway [11], [13], [14], [53]; therefore, it is plausible that in the presence of IL-28 the nominal amounts of IL-10 produced by the CD4^+^ T cells in PLP:OVA-pσ1 + anti-CD25 + anti-TGF-β-dosed mice are in fact proinflammatory. The role for IL-28 has not yet been evaluated in EAE, although IL-28 is known for its anti-inflammatory activity [14], and it has been shown to prime tolerogenic DCs in vitro [12].

In summary, we showed that even a single 100 µg dose of pσ1-based nasal vaccine in an Ag-specific fashion protected mice against EAE. The pσ1-based vaccine protected against EAE via various mechanisms, including activation of IL-10-producing FoxP3^+^ T_reg_ cells and IL-4-secreting FoxP3^+^ Th2 cells. In the absence of T_reg_ cells, pσ1-based protection against EAE was associated with an increased expression of FoxP3 on CD25^−^CD4^+^ T cells producing IL-28, which, to our knowledge, is the first report describing regulatory role of IL-28-producing CD4^+^ T cells conferring protection against EAE. These results show that a single low dose nasal tolerance mediated by genetically modified pσ1 can be successfully applied to prevent and/or treat autoimmune diseases.

## Materials and Methods

### Ethics Statement

All animal care and procedures were in accordance with institutional policies for animal health and well-being, and approved by MSU Institutional Animal Care and Use Committee.

### Preparation of PLP:OVA-pσ1

PLP:OVA-pσ1 was constructed using the OVA-pσ1 backbone [5]. Two copies of PLP peptide (PLP_130–151_; QAHSLERVC HCLGKWLGHPDKF) separated by the flexible linker (RHRHVDCSGRNLTTLPPGLQE) were synthesized as a single cDNA fragment containing restriction enzyme sites 5′ and 3′ termini (GenScript Corp.). The synthetic cDNA fragment was amplified by PCR and cloned into pUC19. The 5′ and 3′ primers encoded *EcoR*I sites, and 5′ primer encoded an ATG initiation codon embedded into an optimal Kozak's sequence. PCR amplified PLP peptides were ligated with the 5′ terminus of OVA-pσ1 in a pPICZ B vector (Invitrogen Corp.) bearing a his-tag carboxy terminus for protein purification (Invitrogen), referred to as PLP:OVA-pσ1. The junction between the PLP_139–151_ epitopes and the OVA-pσ1 featured a flexible linker (Gly-Arg-Pro) to minimize steric hindrance between the components. The resulting construct was sequenced and expressed in the yeast *Pichia pastoris*, according to the manufacturer's directions (Invitrogen Corp.). Recombinant proteins were extracted from yeast cells by a bead-beater (Biospec Products) and purified on a Talon metal affinity resin (BD Biosciences, Palo Alto, CA), according to manufacturer's instructions. Proteins were assessed for purity and quality by Coomassie-stained polyacrylamide gels and by Western blot analysis using a polyclonal rabbit anti-pσ1 (produced in-house) or a polyclonal rabbit anti-OVA Ab (Sigma-Aldrich). All recombinant proteins migrated as a single band with the expected MW.

### Mice

Female six wk old SJL mice were obtained from Frederick Cancer Research Facility, National Cancer Institute, and The Jackson Laboratories. All mice were maintained at Montana State University Animal Resources Center under pathogen-free conditions in individual ventilated cages under HEPA-filtered barrier conditions and were fed sterile food and water *ad libitum*. The mice were free of bacterial and viral pathogens, as determined by antibody screening and histopathological analysis of major organs and tissues.

### Tolerance Induction, PLP:OVA-pσ1 Treatment, and EAE Challenge

For tolerance induction, mice (5–10 mice/group) were nasally dosed up to three times with 50–100 µg of PLP:OVA-pσ1 or OVA-pσ1 before or 6 days after EAE challenge, as described in the text. Control groups were treated with PBS or equivalent amounts of OVA-pσ1. PLP:OVA-pσ1 or OVA-pσ1 was administered nasally, as previously described [5].

For EAE induction, mice were challenged s.c. with 200 µg of the encephalitogenic PLP peptide (PLP_139–151_; HSLGKWLGHPDKF; Global Peptide Services; HPLC-purified to >90%) in 200 µl [7]. On days 0 and 2 post-challenge, mice received i.p. 200 ng of *Bordetella pertussis* toxin (PT; List Biological Laboratories). Mice were monitored and scored daily for disease progression [54]: 0, normal; 1, a limp tail; 2, hind limb weakness; 3, hind limb paralysis; 4, quadriplegia; 5, death.

### Measurement of Delayed-Type Hypersensitivity (DTH) Responses

To measure OVA- or PLP_139–151_- specific DTH responses [55], OVA or PLP_139–151_ (10 µg) was injected into the left ear pinna, and PBS alone (20 µl) was administered to the right ear pinna as a control. Ear swelling was measured 24 h later with an electronic digital caliper (World Precision Instruments). The DTH response was calculated as the increase in ear swelling after antigen injection following subtraction of swelling in the control site injected with PBS.

### Histological Evaluation of Spinal Cords

For histological evaluation of tissue pathology, spinal cords were removed 14 days after challenge and fixed with neutral buffered formalin (VWR International), embedded into paraffin, and sectioned at 5 µm. Cross sections of spinal cords were stained with H&E for pathological changes and inflammatory cell infiltration, and adjacent sections with luxol fast blue (LFB) for loss of myelin. Pathological manifestations were scored separately for cell infiltrates and demyelination. Each H&E section was scored from 0 to 4: 0, normal; 1, cell infiltrate into the meninges; 2, one to four small focal perivascular infiltrates; 3, five or more small focal perivascular infiltrates and/or one or more large infiltrates invading the parenchyma; 4, extensive cell infiltrates involving 20% or more of the white matter [7], [54]. In each LFB stained section, myelin was also scored from 0 to 4: 0, normal; 1, one small focal area of demyelination; 2, two or three small focal areas of demyelination; 3, one to two large areas of demyelination; 4, extensive demyelination involving 20% or more of white matter [7], [54].

### Cytokine ELISA

Spleens, mesenteric lymph nodes (MLNs), and head and neck LNs (HNLNs) were aseptically removed 14 days after EAE induction from PBS-, PLP:OVA-pσ1- and OVA-pσ1-dosed mice. Lymphocytes were prepared, as previously described [7], and resuspended in complete medium (CM) [8]. Lymphocytes were cultured in 24-well tissue plates at 5×10^6^ cells/ml in CM alone or with PLP_139–151_ peptide (30 µg/ml) for 3–5 days at 37°C. The supernatants were collected by centrifugation and stored at −80°C. Capture ELISA was employed to quantify, on triplicate sets of samples, the levels of IFN-γ, IL-4, IL-6, IL-10, IL-13, IL-17, and TGF-β produced by lymphocytes, as previously described [7]. For detection of IL-21, IL-22, and IL-28, microtiter wells were coated with 2 µg/ml of purified goat anti-mouse IL-21 Ab, goat anti-mouse IL-22 Ab, or anti-mouse IL-28B mAb (clone 244716), respectively (all R&D Systems). For detection of IL-23, wells were coated with 8 µg/ml of anti-mouse IL-23p19 (clone G23-8, eBioscience). After blocking with PBS +1% BSA for 2 h at 37°C, washed wells were incubated with cell culture supernatants at 4°C for 24 h. After washing, 0.5 µg/ml biotinylated goat anti-mouse IL-21 Ab, biotinylated goat anti-mouse IL-22 Ab, biotinylated anti-mouse IL-28B mAb (clone 244707) (all R&D Systems), or biotinylated anti-mouse IL-12 and IL-23 (p40) mAb (clone C17.8, eBioscience) was added, respectively, for 90 min at 37°C. Following washing, 1∶500 HRP-goat anti-biotin Ab (Vector Laboratories) was added for 1 h at room temperature (RT). After washing, ABTS peroxidase substrate (Moss, Inc.) was added to develop the reaction. Production of cytokines by unstimulated cells set as a background was subtracted from all measurements.

### FACS Analysis

Lymphocytes from the HNLNs, MLNs, and spleens were isolated 14 days after challenge, and single cell suspensions were prepared, as described above [7]. To obtain lymphocytes from spinal cords, mice were perfused through the left ventricle with 20 ml of ice cold sPBS, and spinal cords were removed by flushing the vertebral canal with media and prepared, as previously described [7].

Cells were stained for FACS analysis using conventional methods. Leukocyte gates were set within the forward and side scatter profiles to exclude resting microglia cells in the spinal cord preparations. Neutrophils and macrophages were analyzed by forward and side scatter profiles and using fluorochrome-conjugated mAbs for SK208 (7,8), CD11b, Gr1, and Mac-3 (BD Pharmingen). T cell subsets were analyzed using fluorochrome-conjugated mAbs for CD4, CD25, TCRβ, CD8, GITR, CCR6 (all from BD Pharmingen), OX-40 (CD134; clone OX-86) (eBioscience), and biotinylated TGF-β(R&D Systems). Intracellular staining for FoxP3 was accomplished using FITC-, Cy-, or PE-anti-FoxP3 mAb (clone FJK-16s; eBioscience), FITC, or PE-anti-IFN-γ Ab, PE, or APC- anti-IL-10 and anti-IL-4, and PE-anti-CTLA-4 (CD152) (all from BD Pharmingen). Bound fluorescence was analyzed with a FACS Canto (BD Biosciences).

### In Vivo Neutralization of IL-4 and Blockade of IL-10R

To inhibit IL-4 in vivo, mice dosed with PLP:OVA-pσ1 on days −14 and −7 before EAE challenge were given i.p. 1.0 mg of anti-IL-4 mAb (clone 11B11; ATCC) on day −1 before challenge, and on day +5 after EAE challenge with PLP_139–151_ [56]. Control mice received i.p. injection of 1.0 mg purified rat IgG Ab (AbD Serotec).

To inhibit IL-10 receptor function, mice were i.p. injected with 0.5 mg of anti-IL-10R mAb (clone1B1.3A, BioXCell), or IgG isotype control Ab at the day of an adoptive transfer and 6 days later (day −1 and +5 relative to the day of EAE induction). Mice were induced with EAE on day 0, as described above.

### In Vitro T Cell Assays

To assess cytokine production by T_reg_ cells and effector T cells, CD25^+^CD4^+^ and CD25^−^CD4^+^ T cells (2×10^5^) were stimulated in vitro with anti-CD3 mAb-coated wells (10 µg/ml; BD Pharmingen) and a soluble anti-CD28 mAb (5 µg/ml; BD Pharmingen) for 5 days (final volume of 300 µl in a 48-well plate). Capture ELISA was used to quantify triplicate sets of samples to measure cytokine production [7].

### Adoptive Transfer Studies

Following PLP:OVA-pσ1 immunization, total CD4^+^ T cells from spleens, HNLNs, and MLNs were obtained (negative CD4^+^ T cell isolation kit, Dynal Biotech ASA). CD25^+^CD4^+^ and CD25^−^CD4^+^ T cells were isolated from total CD4^+^ T cells with >95% and 99% purity, respectively, by positive selection using CELLection Biotin Binder Kit (Dynal Biotech; Invitrogen) and biotin-conjugated anti-mouse CD25 (PC61, eBioscience), according to manufacture's instructions. To test PLP:OVA-pσ1-primed T_reg_ cell efficacy, 6×10^5^ CD25^−^CD4^+^ T cells or CD25^+^CD4^+^ T cells were i.v. injected into naïve recipients. The group of control mice was i.v. injected with sPBS. One day after the adoptive transfer of T cell subsets, mice were challenged with PLP_139–151_ and evaluated for the clinical symptoms.

### In Vivo Inactivation of CD25 and Neutralization of TGF-β and/or IL-28

Mice were nasally dosed with PLP:OVA-pσ1 or PBS on days −14 and −7 relative to the EAE induction with PLP_139–151_. To functionally inactivate CD25^+^CD4^+^ T cells, the same mice were given i.p. 0.5 mg anti-CD25 mAb (clone PC 61.5.3; ATCC TIB-222) on days −5 and −2 before EAE induction. As a control, groups of PLP:OVA-pσ1- or PBS-dosed mice received 0.5 mg of purified rat IgG on the same days before EAE challenge. All mice were monitored daily for development of EAE.

To neutralize TGF-β in vivo, mice dosed with PLP:OVA-pσ1 or PBS and treated with anti-CD25 mAb or rat IgG were i.p. injected with an additional 0.5 mg of anti-TGF-β mAb (clone 1D11.16.8, ATCC) on days −1 and +5 relative to the day of EAE induction.

To neutralize IL-28, mice were i.p. injected on days −3 and +4 relative to the EAE induction with 1 ml of anti-IL-28 RS (developed in-house by immunizing rabbits with recombinant IL-28B; R&D Systems) or with NRS as control.

### In Vivo Neutralization of IL-23

To block IL-23 in vivo, mice dosed with 100 µg of PLP:OVA-pσ1 on day −14 and −7 before EAE challenge were given i.p. 0.5 ml of anti-IL-23p19 rabbit serum (RS, made in-house) on day −1 before challenge, and 0.25 ml of anti-IL-23 RS on days 1 and 5 after EAE challenge with PLP_139–151_. Control mice received i.p. injection of an equal amount of NRS (Jackson ImmunoResearch Laboratories).

### Statistical Analysis

The ANOVA followed by posthoc Tukey test was applied to show differences in clinical scores in treated vs. PBS mice. The student *t* test was used to evaluate the differences between variations in cytokine level production, and P-values <0.05 are indicated, unless specified otherwise.

## Supporting Information

Table S1 Characterization of CD4^+^ T cells from combined LNs and spleens of naïve, PBS- and PLP:OVA-pσ1-dosed SJL mice^a^.
(0.04 MB DOC)

Table S2 In vivo neutralization of IL-4 partially reverses PLP:OVA-pσ1-mediated protection against EAE^a^.
(0.03 MB DOC)

Figure S1 CD25^+^CD4^+^ T_reg_ cells are important for PLP-OVA-pσ1-induced protection against EAE. Mice dosed with PLP:OVA-pσ1 on day 0 and 7, were sacrificed on day 14. CD25^+^CD4^+^ (T_reg_) cells or CD25^−^CD4^+^ (Th2) cells from these mice were adoptively transferred into naive recipients induced with EAE 24 h later. Transfer of T_reg_ cells entirely protected mice from development of EAE, but Th2 cells also significantly delayed and improved severity of EAE. Averaged clinical scores from 2 experiments (10 mice/group) are shown. *, P<0.05 vs. PBS-dosed mice.
(0.18 MB TIF)

Figure S2 IL-4 contributes to PLP:OVA-pσ1-induced protection against EAE. Mice (5/group) dosed with PLP:OVA-pσ1 or PBS on day −14 and −7 were injected with anti-IL-4 mAb or rat IgG on day −1 and +5. CD4^+^ T cells isolated from HNLNs, MLNs, and spleens of these mice at the peak of the disease (day 14 post challenge) were incubated with feeder cells and PLP_139–151_ peptide for 72 h. Cultured supernatants were analyzed for cytokine production by ELISA. Results show cytokine production by cultured CD4^+^ T cells corrected over the cytokine production by unstimulated cells. PBS and PLP:OVA-pσ1-dosed mice treated with anti-IL-4 mAb produced more IFN-γ and less IL-10 than their respective IgG-treated controls. PLP:OVA-pσ1 + anti-IL-4-dosed mice produced less IL-10 and more IFN-γ, IL-6 and IL-17 than PLP:OVA-pσ1 + IgG-dosed mice. PBS + anti-IL-4 -treated mice produced less IL-21 that mice dosed with PBS + IgG. *, P<0.05 for PLP:OVA-pσ1 + anti-IL-4 vs. PBS + IgG or PLP:OVA-pσ1 + IgG, and PBS + IgG vs. PBS + anti-IL-4.
(0.34 MB TIF)

Figure S3 TGF-β is responsible for proinflammatory cytokine production by PLP:OVA-pσ1-tolerized mice depleted of T_reg_ cells. Total lymphocytes isolated from the HNLNs of anti-CD25 and/or TGF-β-treated mice (described in Fig. 5D legend) on day 10 post EAE induction were cultured with PLP_139–151_ peptide for 72 h. PBS-dosed mice independent of Ab treatment produced enhanced amounts of proinflammatory cytokines IL-6, IL-17, IL-21, and IL-23, and little to no IL-4, IL-10, and IL-28. PBS + anti-TGF-β + anti-CD25-treated mice showed elevated IL-22 production. PLP:OVA-pσ1-protected mice treated with IgG, anti-TGF-β mAb or with anti-TGF-β + anti-CD25 mAbs, produced enhanced amounts of IL-4 and IL-28 and little to no of IL-6, IL-17, IL-21, and IL-23. Mean ± SEM of 5 mice per group is shown * P<0.05 for the PLP:OVA-pσ1 + anti-TGF-β + anti-CD25 vs. PLP:OVA-pσ1 + IgG and PLP:OVA-pσ1 + anti-CD25.
(0.74 MB TIF)
