# Supplementary material for: IL-28 Supplants Requirement for Treg Cells in Protein σ1-Mediated Protection against Murine Experimental Autoimmune Encephalomyelitis (EAE)
Source: PLoS One. 2010 Jan 14;5(1):e8720. doi: 10.1371/journal.pone.0008720 (PMC2806841; doi:10.1371/journal.pone.0008720)
Supplement: Table S1 — Characterization of CD4+ T cells from combined LNs and spleens of naïve, PBS- and PLP:OVA-pσ1-dosed SJL micea. (0.04 MB DOC) [file pone.0008720.s001.doc]

**Table S1.**  Characterization of CD4^+^ T cells from combined LNs and spleens of naïve, PBS- and PLP:OVA-pσ1-dosed SJL mice^a^

| **T cells PLP:OVA-pσ1 PBS Naïve** | | | |
| --- | --- | --- | --- |
| **CD25^+^CD4^+^** | 25.10 ± 1.62* | 10.69 ± 1.04 | 5.50 ± 0.68 |
| FoxP3^+^ | 93.10 ± 1.44* | 71.55 ± 2.45 | 43.54 ± 3.72 |
| TGF-β^+^ | 7.09 ± 0.74 | 4.94 ± 1 | 4.42 ± 0.72 |
| GITR^+^ | 19.52 ± 1.3* | 29.91 ± 0.97 | 34.54 ± 2.79 |
| CCR6^+^ | 67.88 ± 1.57* | 25.52 ± 2.21 | 22.26 ± 1.92 |
| CTLA-4^+^ | 48.41 ± 5.34** | 33.03 ± 3.52 | 27.72 ± 2.32 |
| ICOS^+^ | 20.54 ± 1.33 | 22.17 ± 1.81 | 22.44 ± 1.54 |
| IL-10^+^ | 86.35 ± 2.25* | 17.02 ± 1.34 | 9.51 ± 1.19 |
| IL-4^+^ | 7.31 ± 0.88 | 6.29 ± 1.08 | 4.86 ± 1.01 |
| OX-40^+^ | 4.68 ± 0.75 | 4.42 ± 0.96 | 2.58 ± 0.38 |

| **CD25^-^CD4^+^** |  |  |  |
| --- | --- | --- | --- |
| FoxP3^+^ | 19.89 ± 1.08* | 7.84 ± 0.81 | 5.65 ± 0.34 |
| TGF-β ^+^ | 13.44 ± 1.04* | 4.02 ± 0.81 | 1.62 ± 0.42 |
| GITR^+^ | 17.49 ± 1.71** | 11.77 ± 1.45 | 7.36 ± 0.43 |
| CCR6^+^ | 21.42 ± 2.39** | 14.76 ± 1.94 | 9.35 ± 1.18 |
| CTLA-4^+^ | 5.54 ± 0.35 | 6.09 ± 0.8 | 4.41 ± 0.31 |
| ICOS^+^ | 6.51 ± 0.21** | 8.37 ± 0.63 | 8.7 ± 0.86 |
| IL-10^+^ | 6.28 ± 1.11 | 6.98 ± 1.09 | 6.01 ± 0.98 |
| IL-4^+^ | 83.83 ± 2.64* | 4.99 ± 0.54 | 5.98 ± 0.54 |
| OX-40^+^ | 6.85 ± 0.95* | 34.73 ± 1.18 | 2.91 ± 0.5 |

^a^ Mean ± SEM of 10 mice per group is presented. Percentages of CD25^+^CD4^+^ T_reg_ cell and CD25^-^CD4^+^ Th2 cell analyzed by FACS (Figure 2A and B).

*, P < 0.001, ** P < 0.05 for PBS vs. PLP:OVA-pσ1-dosed mice.
