# Supplementary material for: IL-28 Supplants Requirement for Treg Cells in Protein σ1-Mediated Protection against Murine Experimental Autoimmune Encephalomyelitis (EAE)
Source: PLoS One. 2010 Jan 14;5(1):e8720. doi: 10.1371/journal.pone.0008720 (PMC2806841; doi:10.1371/journal.pone.0008720)
Supplement: Table S2 — In vivo neutralization of IL-4 partially reverses PLP:OVA-pσ1-mediated protection against EAEa. (0.03 MB DOC) [file pone.0008720.s002.doc]

**Table S2.**  In vivo neutralization of IL-4 partially reverses PLP:OVA-pσ1-mediated protection against EAE^a^

**Treatment^b^ EAE/Total^c^ Onset^d^ Max. score^e^ CS^f^**

| PBS + IgG | 10/10 | 8.1 ± 0.87* | 5 | 47.2 |
| --- | --- | --- | --- | --- |
| PLP:OVA-pσ1 + IgG | 9/10 | 10.9 ± 1.19 | 2 | 10.4 |
| PBS + anti-IL-4 | 10/10 | 7.7 ± 1.63* | 5 | 62.6 |
| PLP:OVA-pσ1 + anti-IL-4 | 10/10 | 6.6 ± 1.07* | 5 | 40.4 |

^a^ SJL/J mice were challenged s.c. with 200 μg PLP_139-151_ in complete Freund’s adjuvant plus 200 ng PT i.p. on days 0 and 2.

^b^ Mice were nasally immunized 14 and 7 days prior to EAE challenge with 100 μg of PLP:OVA-pσ1 or with PBS (Figure 3C)

^c^ Number of mice with EAE/total in group.

^d^ Mean day ± SD of clinical disease onset.

^e^ Maximum (Max.) daily clinical score.

^f^ Cumulative scores (CS) were calculated as the sum of all scores from disease onset to day 26 post-challenge, divided by the number of mice in each group. *, P < 0.001 **, P < 0.05 for PBS vs. PLP:OVA-pσ1-dosed mice.
