# Supplementary figures and images for: IL-28 Supplants Requirement for Treg Cells in Protein σ1-Mediated Protection against Murine Experimental Autoimmune Encephalomyelitis (EAE)
Source: PLoS One. 2010 Jan 14;5(1):e8720. doi: 10.1371/journal.pone.0008720 (PMC2806841; doi:10.1371/journal.pone.0008720)

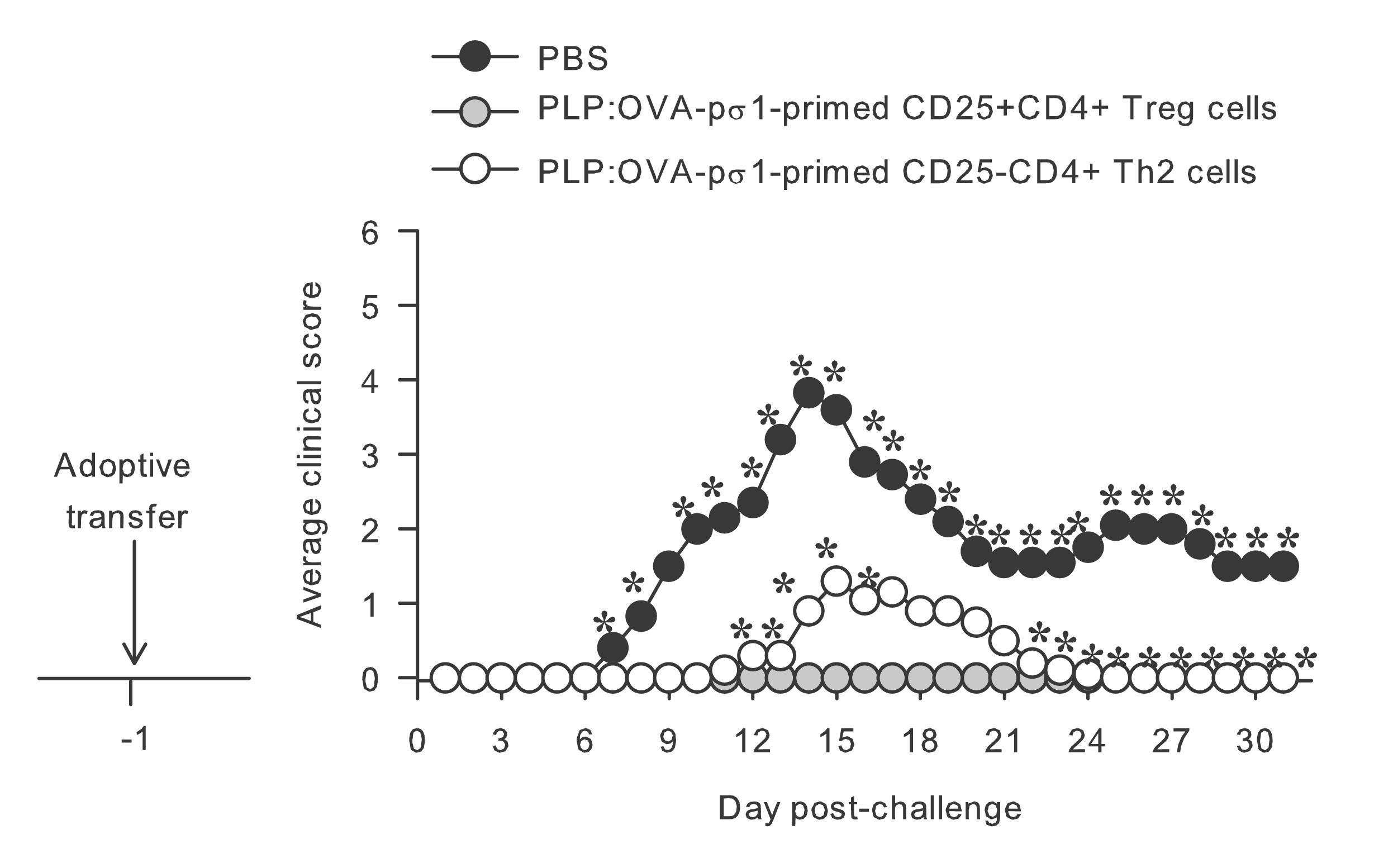

Supplement: Figure S1 — CD25+CD4+ Treg cells are important for PLP-OVA-pσ1-induced protection against EAE. Mice dosed with PLP:OVA-pσ1 on day 0 and 7, were sacrificed on day 14. CD25+CD4+ (Treg) cells or CD25−CD4+ (Th2) cells from these mice were adoptively transferred into naive recipients induced with EAE 24 h later. Transfer of Treg cells entirely protected mice from development of EAE, but Th2 cells also significantly delayed and improved severity of EAE. Averaged clinical scores from 2 experiments (10 mice/group) are shown. *, P<0.05 vs. PBS-dosed mice. (0.18 MB TIF) [file pone.0008720.s003.tif]

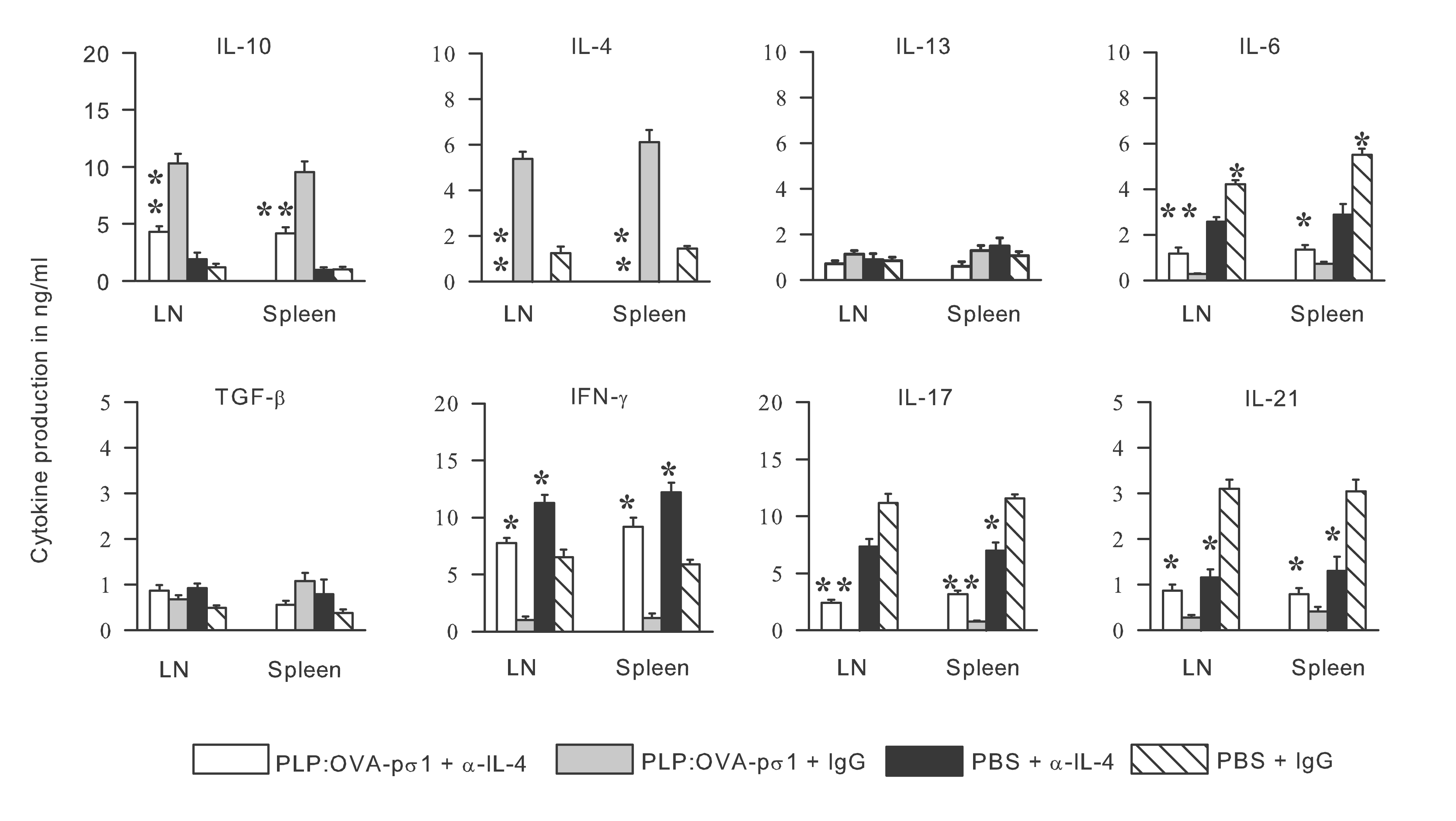

Supplement: Figure S2 — IL-4 contributes to PLP:OVA-pσ1-induced protection against EAE. Mice (5/group) dosed with PLP:OVA-pσ1 or PBS on day −14 and −7 were injected with anti-IL-4 mAb or rat IgG on day −1 and +5. CD4+ T cells isolated from HNLNs, MLNs, and spleens of these mice at the peak of the disease (day 14 post challenge) were incubated with feeder cells and PLP139–151 peptide for 72 h. Cultured supernatants were analyzed for cytokine production by ELISA. Results show cytokine production by cultured CD4+ T cells corrected over the cytokine production by unstimulated cells. PBS and PLP:OVA-pσ1-dosed mice treated with anti-IL-4 mAb produced more IFN-γ and less IL-10 than their respective IgG-treated controls. PLP:OVA-pσ1 + anti-IL-4-dosed mice produced less IL-10 and more IFN-γ, IL-6 and IL-17 than PLP:OVA-pσ1 + IgG-dosed mice. PBS + anti-IL-4 -treated mice produced less IL-21 that mice dosed with PBS + IgG. *, P<0.05 for PLP:OVA-pσ1 + anti-IL-4 vs. PBS + IgG or PLP:OVA-pσ1 + IgG, and PBS + IgG vs. PBS + anti-IL-4. (0.34 MB TIF) [file pone.0008720.s004.tif]

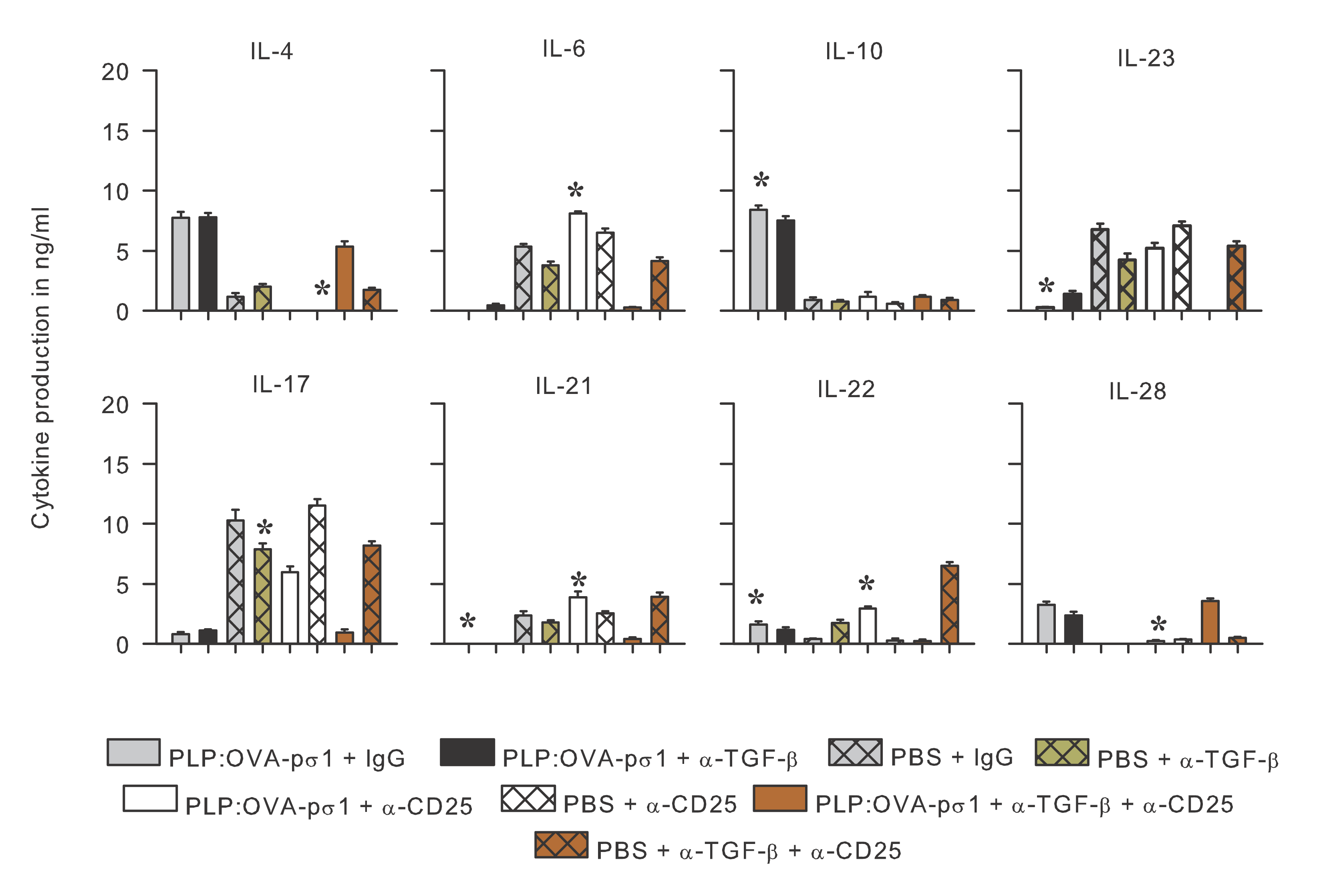

Supplement: Figure S3 — TGF-β is responsible for proinflammatory cytokine production by PLP:OVA-pσ1-tolerized mice depleted of Treg cells. Total lymphocytes isolated from the HNLNs of anti-CD25 and/or TGF-β-treated mice (described in Fig. 5D legend) on day 10 post EAE induction were cultured with PLP139–151 peptide for 72 h. PBS-dosed mice independent of Ab treatment produced enhanced amounts of proinflammatory cytokines IL-6, IL-17, IL-21, and IL-23, and little to no IL-4, IL-10, and IL-28. PBS + anti-TGF-β + anti-CD25-treated mice showed elevated IL-22 production. PLP:OVA-pσ1-protected mice treated with IgG, anti-TGF-β mAb or with anti-TGF-β + anti-CD25 mAbs, produced enhanced amounts of IL-4 and IL-28 and little to no of IL-6, IL-17, IL-21, and IL-23. Mean ± SEM of 5 mice per group is shown * P<0.05 for the PLP:OVA-pσ1 + anti-TGF-β + anti-CD25 vs. PLP:OVA-pσ1 + IgG and PLP:OVA-pσ1 + anti-CD25. (0.74 MB TIF) [file pone.0008720.s005.tif]
